# Old and New Aphid-Borne Viruses in Coriander in Chile: An Epidemiological Approach

**DOI:** 10.3390/v16020226

**Published:** 2024-01-31

**Authors:** Alan Zamorano, Paulina Carevic, Camila Gamboa, Weier Cui, Tomislav Curkovic, Pamela Córdova, Gastón Higuera, Luz Ramos-Castillo, Nicolás Quiroga, Nicola Fiore

**Affiliations:** 1 Facultad de Ciencias Agronómicas, Universidad de Chile, Avenida Santa Rosa 11315, Santiago 8820808, Chile; agezac@uchile.cl (A.Z.); p.carevicelgueta@gmail.com (P.C.); camila.gamboa@uchile.cl (C.G.); cuiweierpku@gmail.com (W.C.); tcurkovi@uchile.cl (T.C.); luzramoscast@uchile.cl (L.R.-C.); 2 Instituto de Nutrición y Tecnología de los Alimentos, Universidad de Chile, Avenida El Líbano 5524, Santiago 7830490, Chile; pamela.cordova@inta.uchile.cl (P.C.); gastonhiguera@inta.uchile.cl (G.H.); 3 Institute of Agri-Food, Animal and Environmental Sciences (ICA3), Universidad de O’Higgins, Campus Colchagua, San Fernando 3070000, Chile; nicolas.quiroga@uoh.cl

**Keywords:** HTS, coriander, insect vector, *Potyviridae*, *Solemoviridae*, *Rhabdoviridae*, aphids

## Abstract

In Chile, edible herbs are mainly grown by small farmers. This type of horticultural crop typically requires intensive management because it is highly susceptible to insects, some of which transmit viruses that severely affect crop yield and quality. In 2019, in coriander plants tested negative for all previously reported viruses, RNA-Seq analysis of one symptomatic plant revealed a plethora of viruses, including one virus known to infect coriander, five viruses never reported in coriander, and a new cytorhabdovirus with a 14,180 nucleotide RNA genome for which the species name *Cytorhabdovirus coriandrum* was proposed. Since all the detected viruses were aphid-borne, aphids and weeds commonly growing around the coriander field were screened for viruses. The results showed the occurrence of the same seven viruses and the alfalfa mosaic virus, another aphid-borne virus, in aphids and weeds. Together, our findings document the presence of multiple viruses in coriander and the potential role of weeds as virus reservoirs for aphid acquisition.

## 1. Introduction

Chilean agriculture is widely known for its high-quality fruit production, being one of the most important fruit exporters in the world. On the other hand, the local production of vegetable crop is primarily directed to the domestic market. In 2018, the total area used for vegetable production in Chile reached 91,000 hectares, with species from the Solanaceae and Brassicaceae families being among the most important crops [1]. On the country, more than 30% of the cultivated area is located around the city of Santiago, in the metropolitan region, where more than 40% of the population lives. Even though the urban area is continuously growing, with a significant loss of cultivable land, peri-urban family farming is still one of the most important rural activities [2]. The historically favorable climatic conditions in the central zone of Chile have led to the production of a wide variety of vegetables grown together in these peri-urban areas [3].

Among the many cultivated crops, coriander (*Coriandrum sativum* L.) is of medium importance, reaching 787 hectares of production and being widely distributed throughout the country [4]. Many pathogens have been reported to affect this crop, such as *Pseudomonas syringae* pv. *coriandricola*, *Ramularia coriandris*, *Fusarium oxysporum*, *Helminthosporium* spp., and *Curvularia* spp., as well as diseases caused by viruses [5]. In Chile, coriander has been reported to be affected by fungal pathogens such as *Fusarium* spp. and *Cercospora coriandri*, among others, or bacterial pathogens such as *Pseudomonas cichorii*, but there is no information on viral diseases [6].

Viruses greatly affect the yield and quality of their hosts, shortening their productive lifespan, and making them more susceptible to other infectious agents [7]. Several insect-borne viruses have been detected in coriander plants worldwide, such as the coriander feathery red-vein virus (CFRVV), alfalfa mosaic virus (AMV), celery mosaic virus (CeMV), apium virus Y (ApVY), carrot virus Y (CaV-Y), carrot red leaf virus (CaRLV), and carrot mottle virus (CaMoV) transmitted by aphids [8,9,10,11]; the groundnut ringspot virus transmitted by thrips [12]; and the lettuce chlorosis virus and beet pseudo-yellows virus transmitted by whiteflies [13]. Of these, only the AMV and CeMV have been reported in Chile in other crops [5].

**Figure 1:**
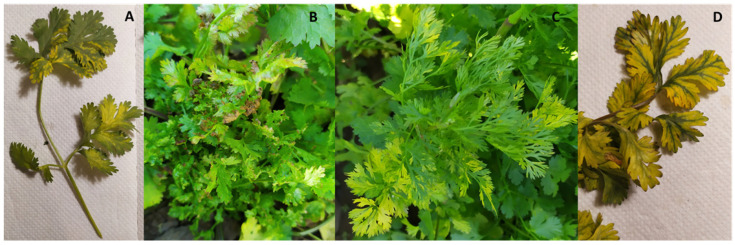
Symptoms associated with infected coriander plants. (**A**) Mosaic on leaflets, (**B**) rosettes, (**C**) leaf yellowing, (**D**) red veins.

During the summer of 2019, several small farmers from the western side of the metropolitan region (Chile), in the municipality of Maipú, observed a wide variety of symptoms in coriander plants, ranging from rosettes, reddening of the veins, complete yellowing, and mosaics (Figure 1). In the present study, symptomatic coriander plants were analyzed by RT-PCR for the detection of some of the above-mentioned viruses. In parallel, a high throughput sequencing (HTS) approach was used to identify the virome of symptomatic coriander plants. Based on the HTS results, a small-scale survey of viruses in aphids and weeds within or surrounding the coriander fields was performed. Here are reported our findings of the occurrence of a virus known to infect coriander, five viruses reported for the first time in coriander, and a new virus in coriander.

## 2. Materials and Methods

### 2.1. Plant and Insect Material

Coriander plants showing symptoms of possible viral etiology were collected from two fields located in Rinconada de Maipú, Santiago de Chile. In addition, symptomatic leaves of several weed species were collected in the immediate vicinity of the coriander fields. Photographs were taken for morphological identification, and the plants were transferred to the laboratory in plastic bags in a cooler. For plant identification, the morphological characterizations described by [14,15,16] were used for reference. Between five to fifteen aphids were collected from each plant using the shoveling or vegetative shoot collection method (in the case of the presence of aphid colonies). The aphids were transported alive in labeled vials. For their morphological identification, photographs of the individuals were taken through a stereoscopic magnifying glass. In addition, the morphological characters were photographed to achieve the identification of each species [17]. For this purpose, a matrix of morphological characters was developed in which the following were observed, compared, and analyzed: body length (millimeters), body shape (oval, elongated, or spindle-shaped), body color (greenish, yellowish, etc.), eye color (dark brown, red, or black), approximate length of antennae (millimeters), approximate length of legs (millimeters), color of leg segments (dark, light, etc.), length of siphons in relation to body, shape of siphons (conical, cylindrical, subcylindrical, etc.), color of siphons (dark, light, or light with darkened tip), and shape of tail (papilla with rounded anal plate, papilla, triangular, digitiform, or linguliform) [18]. It should be noted that five adult individuals per sample were used to obtain these characters and that the length measurements (total body, antennae, legs, and siphons) were made from previously preserved individuals in 70% ethanol.

Morphological identification was conducted based on the information provided in the character matrix and morphological characterizations described by [19,20,21,22,23].

### 2.2. High Throughput Sequencing and Bioinformatic Analysis on Coriander

Total RNA was extracted from symptomatic leaves of a single coriander plant using a Sigma Spectrum™ Plant Total RNA extraction kit (MilliporeSigma, Burlington, MA, USA) and was sent to Psomagen Inc. for RNA-Seq using the Illumina Novaseq 6000 platform with a read length of 150 bp paired ends. The library was constructed using TruSeq Stranded Total RNA with Ribo-Zero Plant in order to maximize viral RNA output. Raw reads were trimmed and assembled using CLC Genomics Workbench v24.1. Contigs were compared against public databases (BLAST and VirFind) and also against an internal custom database using the CLC Genomics Workbench v24.1 BLAST tool.

Alignments and phylogenetic reconstructions were performed using CLC Genomics Workbench and MEGA v7.0 [24], using neighbor-joining and maximum-parsimony algorithms.

### 2.3. Small-Scale Survey

#### 2.3.1. RNA and Total Nucleic Acid Extraction

Total RNA was extracted from leaf tissues of weeds using a Spectrum™ Plant Total RNA kit (MilliporeSigma, Burlington, MA, USA) according to the manufacturer’s instructions. In addition, all plants were processed for total nucleic acid extraction using the modified silica-capture method described by [25]. The silica-capture method was also used for RNA extraction from insects, with a modification of the volume of the grinding buffer in the first step. Eight to ten insects were used for RNA extraction with an initial volume of 0.3 mL of grinding buffer instead of the usual volume of 1.5 mL used for plant extracts.

For the plants, approximately 150 mg of fresh tissue per sample was used as the starting material, and for insects, three individuals from each species were used per sample.

#### 2.3.2. RT-PCR Virus Detection

Two-step RT-PCR was performed using random primers (random hexanucleotide DNA, Roche, Basel, Switzerland) and MMLV-RT (Promega, Madison, WI, USA) for reverse transcription, followed by standard Taq polymerase amplification (Invitrogen, Thermofisher, Waltham, MA, USA).

A total volume of 30 µL was used for PCR amplification, including a 2.5 µL cDNA and 27.5 µL amplification mix consisting of 1 µL of d-NTP, 1 µL of each primer, 1.5 µL of MgCl2, 0.2 µL of Taq DNA Polymerase (Invitrogen, Sao Paulo, Brazil), 3 µL of buffer, and 21 µL of sterile water. The amplification program was specific for each virus. The primers used for the detection of each virus were designed based on the genomic sequences obtained by HTS analysis and are described in Table 1. The amplification products were separated by 1.2% agarose gel electrophoresis, and the purified PCR products were sequenced at Psomagen Inc. (Rockville, MD, USA).

#### 2.3.3. Molecular Identification of Aphids

The morphological identification described above was complemented by a molecular analysis using the collected individuals, which were labeled and stored in Eppendorf tubes with 70% ethanol. The molecular analysis was performed by amplification of the mitochondrial gene cytochrome C oxidase subunit I (COI), known as “DNA barcode”, using the same total nucleic acid extract previously described for insects and the universal PCR primers LCO1490 (5′-GGTCAACAAATCATAAAGATATTGG-3′) and HCO2198 (5′-TAAACTTCAGGGTGACCAAAAAATCA-3′) [30]. The amplicons were directly sequenced at Psomagen Inc., USA, and the sequences were compared with those in public databases such as GenBank and the DNA Barcode Genetic Database (http://www.barcodinglife.org/). Alignment with the reference sequences was performed using Bio-edit v7.2 software, and phylogenetic construction was performed using neighbor-joining and maximum likelihood methods using the MEGA v7.0 program.

## 3. Results

### 3.1. High Throughput Sequencing and Bioinformatic Analysis on Coriander

A total of 57,679,195 raw reads were generated from the total RNA sequencing. Illumina reads were trimmed for quality and length to perform de novo assembly with a minimum contig size of 200 bp in length. A total of 93,071 contigs were obtained with an N50 of 1696 and an N75 of 749 bp. The contig sequences were compared with those in public databases (BLAST and VirFind) and with those in an internal CLC virus database using the Blastn and tBlastx algorithms. This analysis led to the identification of virus-specific contigs. In particular, the complete genome of the celery mosaic virus (CeMV, 10,026 nt; GenBank accession number OR536953) was obtained, as well as the complete coding sequences of other viruses never reported in coriander: the beet mosaic virus (BtMV, 9592 nt; GenBank accession number OR536954), turnip yellows virus (TuYV, 5679 nt; GenBank accession number OR536957), and artichoke latent virus (ArLV, 8536 nt; GenBank accession number OR536955). But the most surprising result was a long contig with high coverage showing identity matches with viruses belonging to the Rhabdoviridae family: a putative member of the genus Cytorhabdovirus, 14,180 nt in size (GenBank accession number OR536958). In addition, another virus, the potato leafroll virus, was not completely assembled from the sequencing reads (5169 nt), so the complete coding sequence of the virus was obtained by RT-PCR and Sanger sequencing using the primers described in Table S1. The complete sequence of the PLRV reached a total of 5787 nt (GenBank accession number OR536956). Finally, partial fragments of a member of the genus Umbravirus, the opium poppy mosaic virus, were detected, but it was not possible to obtain the full sequence, so the assignment of the GenBank accession number is pendent. Neighbor-joining phylogenetic analyses performed on the full genomes of the detected viruses confirmed the taxonomic identification (Supplementary Figures S1–S3).

### 3.2. Molecular Characterization of the New Coriander Cytorhabdovirus

**Figure 2:**
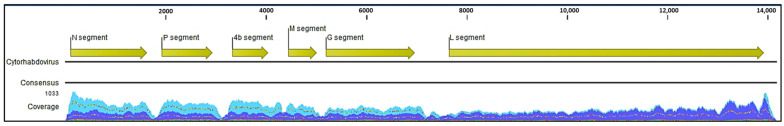
Genomic distribution of coriander cytorhabdovirus 1.

The contig of the tentative new cytorhabdovirus, of 14,180 nt, showed a total coverage of 776.43x. The highest Megablast matches with members of the genus Cytorhabdovirus. The complete replicase gene was amplified by PCR using primers indicated in Table S1, sequenced, and aligned with the complete sequence obtained by HTS, with all fragments matching the assembled sequence at 100% nucleotide identity (GenBank accession number OR536958). The complete genome contained six predicted ORFs (Figure 2), which were identically organized as the typical cytorhabdovirus genomic structure [31]. Looking at the positive-sense genomic RNA, the first gene encoded a nucleocapsid protein (pfam03216) of 480 amino acids and shared the highest identity with the N segment of the raspberry vein chlorosis virus RVCV (52.76%). The second ORF encoded a putative phosphoprotein of 325 amino acids with 45.25% identity to the RVCV phosphoprotein. ORF3 encoded a conserved movement protein known as “4b protein” with a size of 235 amino acids and reaching 59.46% identity with the same protein of the RVCV. ORF4 encoded the rhabdovirus conserved matrix protein (M segment) of 184 amino acids in length, which shared 53.59% identity with the M protein of the RVCV. ORF5 encoded the conserved rhabdovirus glycoprotein, which was 571 amino acids long and had a maximum identity of 53.36% with the RVCV glycoprotein. Finally, ORF6 encoded a 2092 amino acid L-replication protein that included the conserved domains of the Mononegavirales RNA-dependent RNA polymerase (pfam00946) and Mononegavirales mRNA-capping region V (pfam14318), both found in all replication proteins from viral species of the family Rhabdoviridae. The protein showed a high identity with the RVCV L-protein (65.39%) and exhibiting the conserved motifs found in the replicase protein of all the members of the Rhabdoviridae family (Figure S4). Based on this information, we tentatively named this pathogen as coriander cytorhabdovirus 1.

**Figure 3:**
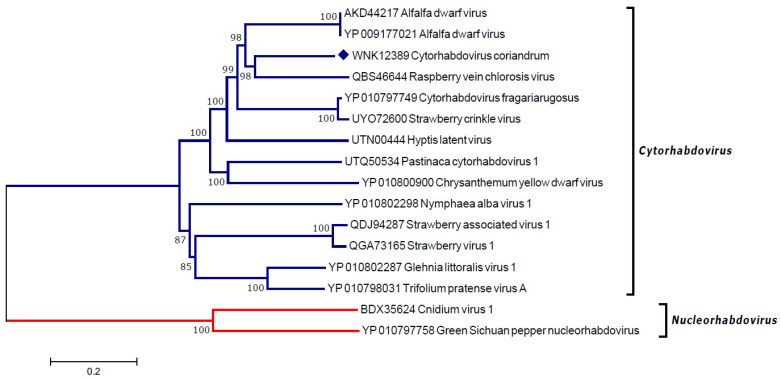
Phylogenetic analysis of coriander cytorhabdovirus 1 with other members of the *Rhabdoviridae* family. The tree was constructed using deduced amino acid sequences of the replicase gene. Blue branches were used for taxa belonging to the genus *Cytorhabdovirus*. Red branches were used for outgroup taxa, belonging to genus *Nucleorhabdovirus*.

The replicase gene sequence was used for the validation of the detection and also to construct a phylogenetic tree using the deduced amino acid sequence. Figure 3 shows the clustering of the coriander cytorhabdovirus 1 isolate within the branch formed with isolates of different species of the genus Cytorhabdovirus.

### 3.3. Identification of Potential Alternative Hosts and Aphids

A total of 38 plant samples (coded P1-P38) with virus symptoms were collected in three sampling periods from September 2020 to January 2022. In the first sampling (M1), 10 herbaceous plants other than coriander were collected in the surrounding area of the coriander field where the original HTS-analyzed plant was collected (C1), with the coriander being already harvested at that time. In the second sampling event (M2), 19 herbaceous plants were collected from four different adjacent fields (C1–C4), again including samples from plot C1, which at that time had an established potato (*Solanum tuberosum* L.) field. Spontaneous plant samples of *Sonchus olearaceus* and alfalfa (*Medicago sativa* L.) were collected from C2. Samples of *S. oleraceus*, *Chenopodium* sp. and *Brassica rapa* were collected from C3, and samples from an established coriander crop were collected from C4, where most of the plants showed symptoms associated with viral diseases. In the third and final sampling period (M3), samples of coriander and mallow were collected from a fifth plot (C5) and a sample of basil (*Ocimum basilicum* L.) was collected from the adjacent field (C6). Samples from plots C1 and C3 were also included.

In addition to the collection of plants, insect samples, mainly aphids, were also collected, considering those associated with the sampled plants, with the purpose of evaluating the presence of the coriander-associated viruses and analyzing their role as potential vectors of said viruses. A total of 29 specimen samples (coded as A1–A28 and A20.1) were collected during the first (M1) and second (M2) plant sampling periods (each sample corresponded to a colony of approximately five to fifteen individuals). In the first period, 10 samples were collected, and in the second period, 19 samples were collected (Supplementary Table S2). During the third sampling period (M3), due to the warm and dry Chilean summer season, no aphids were observed in the collected plants.

To complement the morphological identification of the collected insects, molecular analyses were performed on the 29 samples (colonies). Supplementary Table S2 summarizes the results of the identification of the collected plants and their associated insects. In most cases, a good agreement was observed between the morphological and molecular identification of the analyzed insects. As shown in Table S2, in the cases of *Chaitophorus leucomelas* (A12) and *Cavariella aegeopodii* (A22), their morphological identifications could not be determined. In addition, for the samples A2 and A20.1, two specimens of lace bugs, insects belonging to the suborder Heteroptera, the morphological analysis based on the character matrix was not performed and the molecular identification showed 86.8% nucleotide identity with *Corytucha padi*. In the cases of A26 and A27, there were between two and three winged individuals per sampled plant, so they were not analyzed morphologically. In addition, A26 and A27 did not present results in the molecular analysis due to a very low-quality sequence, probably because the aphids were parasitized.

### 3.4. RT-PCR Virus Detection in Plants and Insects

A total of 38 plants were tested by RT-PCR for the detection of the AMV, and the six viruses identified by HTS were the BtMV, TuYV, Cytorhabdovirus, ArLV, PLRV, and CeMV. Among them, 32 were positive for at least one virus. As shown in Table 2, single infections were found in 18 plants; 5 were positive for the TuYV, and 13 plants were infected with the AMV. On the other hand, mixed infections were also abundant, with double infections in eleven plants, triple infections in two plants, and one plant simultaneously infected with four viruses.

On the other hand, of the 29 insect samples analyzed, 17 were positive for at least one virus with 14 samples containing one virus, one sample containing two viruses, and 2 samples containing three viruses.

## 4. Discussion

This work initially focused on detecting the presence of viruses that could explain the symptoms observed in coriander fields. HTS revealed the occurrence of several viruses, including the CeMV that is known to infect coriander, five other viruses reported for the first time in coriander (the BtMV, TuYV, PLRV, ArLV, and OPMV), and a new cytorhabdovirus in the family *Rhabdoviridae*. To our knowledge, a previous study identified the coriander feathery red-vein virus (CFRVV), a member of the *Rhabdoviridae* family that infects coriander [32]. In their study, Misary and Sylvester showed that the aphid-borne CFRVV caused characteristic symptoms in coriander and was transmitted by the aphid *Hyadaphis foeniculi*. Although some of the symptoms observed on Chilean coriander plants were similar to those described for the CFRVV (e.g., reddening of the veins), we were unable to find a plant with a single cytorhabdovirus infection. In addition, since the identification of the CFRVV occurred in 1983, there is no genomic information in the public databases, so we could not assume that we were dealing with the same virus. Nevertheless, this is the first report of a member of the genus *Cytorhabdovirus* infecting coriander in Chile or anywhere in the world.

Regarding the identification of multiple viruses in a single sample of a coriander plant, it is worth mentioning that multiple virus infections have been previously reported in coriander plants, so this phenomenon would not be an isolated case, although it is surprising due to the high number of viruses simultaneously identified in a single plant (seven viruses). In previous reports, such as [13], the authors detected the co-infection of the alfalfa mosaic virus (AMV) and lettuce chlorosis virus (LCV) or the beet pseudo-yellows virus (BPYV) in coriander plants. This finding, together with what was observed in this study, suggested that the number of viruses capable of infecting (or co-infecting) coriander and/or our ability to detect and identify them is increasing thanks to advanced HTS techniques.

In addition to the studies mentioned above, other studies [33,34] also reported the infection of coriander plants by viruses previously described in other plant species. This was the case for the carrot virus Y (CarVY), which was shown to infect not only carrots but also other plants, including anise, chervil, coriander, cumin, and dill [34]. In this case, the authors mentioned that remnants of native vegetation, which may include native apiaceous species, sometimes occurred within carrot growing areas and could thus act as a reservoir for the CarVY. In addition, the watermelon mosaic virus (WMV) was shown to cause leaf mottling in coriander [33]. It is important to emphasize that coriander has previously been identified as a symptomless host of the WMV, acting as a potential inoculum source for cucurbits, but their study was the first to show that the WMV caused leaf mottling disease in coriander plants. This situation tells us that, in addition to our ability to detect viruses using HTS techniques, different viruses are acquiring infectious capabilities on new hosts, often sharing a physical space or found in adjacent crops. This suggests that something similar may be happening in several horticultural crops, including coriander, where weed and insect management is poor and may help to increase the risk of virus movement from alternative hosts (reservoirs) by insect vectors (aphids).

The green peach aphid, *Myzus persicae*, has been shown to be capable of naturally transmitting the CeMV, BtMV, ArLV, TuMV, and PLRV. Even though this aphid is widespread in Chile, we cannot disregard that these viruses have evolved to be efficiently transmitted by other aphid species. Regarding rhabdoviruses, it is known that some of them are transmitted by aphids, planthoppers, or leafhoppers, situating them together with the known viruses in the possibility of a non-persistent transmission by aphid species. Previous reports have indicated that in the last decade, *M. persicae* and other aphids have started to migrate as much as two weeks earlier in temperate zones worldwide, delaying the dates of final migrations and showing an increase in the number of reproductive cycles per season. This behavior, especially of poikilothermic insect species, favors the spread of viruses. For this reason, the increase in the global average temperature has become more important, since in the last few years, several diseases related to viruses and other virus-like diseases have been observed in vegetables. Therefore, to ensure a successful harvest, it is essential to correctly identify and control insects to prevent the spread of pathogens.

The identification of aphids is considered a difficult task due to their morphological plasticity and sometimes their lack of clearly differentiated morphological characters, since they have a large amount of intraspecific variation and phenotypic similarity between different species, which could prevent an adequate identification. However, most of the samples morphologically analyzed in this study matched with the molecular identification. In recent years, the usefulness of the DNA barcoding method (DNA barcode) has demonstrated its value to complement and support the identification of these species. In aphids, it has been found that a threshold of 2% genetic distance is sufficient to distinguish most species [35]; therefore, upon obtaining an identity percentage of ≥98% similarity, it can be said that it is the same species. Of the 29 aphid samples analyzed, 21 coincided with an identity percentage of ≥98% with the previous identification based on the morphological matrix, supporting the idea that the DNA barcoding method is a solid method to quickly and accurately identify species, being a good complementary tool to morphological identification, especially for cryptic species such as aphids.

Among the criteria for the identification of a virus, the mode of transmission and the identification of the vector are some of the most important factors to be considered since they determine its ability to spread and show us the possible means of control that can be applied [36]. In this study, all the positive plants harbored viruses with this transmission mechanism, which was exclusively associated with aphid vectors. Acquisition and inoculation occurred during the brief (seconds to minutes) aphid feeding, making the spread much faster and therefore difficult to control. This characteristic may have explained why some of these viruses were not detected by molecular analysis in the aphids analyzed, but rather in the plants that harbored them. Nevertheless, our future research will focus on establishing transmission assays to determine if the aphid species detected as positive for the viruses are capable of transmitting the virus to plant hosts.

Focusing specifically on the new cytorhabdovirus, during this small survey, we detected only a single weed plant (*S. oleraceous*) infected with the new virus. This plant was collected in the same fields where we collected the HTS-analyzed coriander plant. As pointed out by [36], members of the *Rhabdoviridae* family could be transmitted in a persistent–circulative mode. In this mode of transmission, the virus is absorbed and retained in the tissues of the insect and is characterized by an invasion of the salivary glands. Viruses must be able to cross the insect’s gut and spread to adjacent organs to reach the salivary glands for transmission. This action requires a latency period within the vector and requires crossing the insect’s blood–brain barrier. This activity involves complex interactions between the transmitted viruses and their insect vectors, and the specificity of virus–vector interactions is mainly determined by the viral capsid (proteins and glycoproteins) as well as some non-structural proteins.

Like other viruses that replicate in their vectors, rhabdoviruses have a latency period of 3 to more than 60 days [37], which means that their spread can be extended and maintained over time. They are commonly transmitted by aphids and leafhoppers (Hemiptera: Cicadellidae), among other arthropods [38]. In this study, the cytorhabdovirus was detected in an aphid of the species *Hyperomyzus lactucae*. This aphid is known to transmit two rhabdoviruses, the lettuce necrotic yellow cytorhabdovirus (LNYV) [39] and sowthistle yellow vein virus (SYVV). According to [38], the latency period of the SYVV in *H. lactucae* was long and highly dependent on the ambient temperature. The reasons for this much longer latency period compared to simply circulating (non-propagating) viruses are probably related to the need for the virus to replicate before it becomes transmissible, giving it relevant phytopathological importance compared to other transmission mechanisms.

The insect samples A2 and A20.1, identified as members of the suborder Heteroptera, presented 86.8% nucleotide identity with *Corythucha padi* COI sequences. In Chile, there are five described species [40,41] of this group of insects commonly called “lace bugs” (Hemiptera, Tingidae). Among them, only one corresponds to the genus *Corythucha*, the species *Corythucha ciliata*. Some species of the suborder Heteroptera have been previously reported as vectors of viruses in plants, such as *Piesma quadratum* (Hemiptera, Heteroptera, Piesmatidae), which is a vector of beet leaf curl virus (BLCV), a member of the family *Rhabdoviridae* [42]. Although rhabdoviruses were not detected in A2 and A20.1 insects on this occasion, the potential of *Corytucha* sp. as a vector also warrants further study.

It has been noted that RNA viruses are usually subjected to high mutation rates, short replication times, and large population sizes to ensure their survival over time, thus having a high evolutionary potential that makes them the main pathogens responsible for emerging diseases [43]. Viruses generate the ability to adapt to the different characteristics of the environment, thus adapting to new hosts to maintain their persistence, even more so in the absence of their primary hosts, generating new variants and interactions that ensure their stay and development, managing to expand and spread throughout the world in the long-term. For this reason, and because it is very difficult to eradicate these pathogens once they are established, it is important to know how they behave in the field, which involves their possible hosts and/or reservoir plants and possible insect vectors. Knowing these parameters, we can develop a preventive management plan that takes these factors into account, with a series of decisions that can reduce the impact of an infection on our plants in the future, and therefore on our production. These actions can make a big difference in the local economy, especially in small-scale agriculture.

## 5. Conclusions

This study provided novel information about the viruses affecting coriander, their potential aphid vectors, and their reservoir plants. However, due to the large number of viruses co-infecting the coriander plants, it was not possible to associate symptoms with each virus species detected.

Epidemiological studies were conducted to identify the potential insect vectors and reservoir plants of the detected viruses. Based on the obtained results, it was determined that weed reservoir plants (*B. rapa*, *Chenopodium* sp., *U. urens*, *Epilobium* sp., *Amaranthus* sp., *M. nicaeensis*, *S. oleraceus*, and *A. retroflexus*) were identified for the viruses TuYV, OPMV, AMV, and a new cytorhabdovirus, all of which were detected in coriander plants by HTS. It should be noted that among the viruses detected, the BtMV, TuYV, and OPMV had not been previously reported in coriander plants anywhere in the world.

According to our results, the ArLV, TuYV, PLRV, coriander cytorhabdovirus 1, OPMV, and AMV were detected in aphids of the species *B. brassicae*, *M. euphorbiae*, *M. carnosum*, *A. solani*, *H. lactucae*, *Aphis nerii*, *M. persicae*, *Cavariella* sp., and in the bug *Corytucha* sp. This result indicated the need to carry out transmission assays in order to effectively determine whether or not insects were the vectors of the said viruses.

Our work confirmed the importance of epidemiological studies to decipher the potential role of insects and plants as viral reservoirs and their contribution to the spread of and difficulty in the eradication of crop-associated viruses.

## Figures and Tables

**Table 1:** Primer pairs used for virus detection.

Virus	Genomic Region	Sequence (5′-3′)	Amplicon Size	References
ArLV	PC4	GGCAAATCCTTCAACAGGGA ATCACATGAGCGGGCATTA	660 bp	This work
TuYV	RdRp	CGTAAAAGCAATCAAAGAGC TCATACAAACATTTCGGTGTAGAC	633 bp	This work; [26]
BtMV	CP	AATGCGCAACAGAGAAAG TCTCTGTATCCTCCGATGT	226 bp	This work
CeMV	Nib	GGTGGTTTTGGCAATGACGT GCTGGTTCACTTGATCGATCC	317 bp	This work
Cytorh	GP	TGGTTGTGATCAGTTTGATGAG AACATATGTCCGAACTCAATTTCA	290 bp	This work
OPMV	RdRP	CGGTGTCCACAACAACTC GGCATGGTTCGTGTACATC	645 bp	[27]
PLRV	CP	CCACTCCAACTCCCCAGAAG TACATAGGGACGGCTTGCAT	208 bp	[28]
AMV	CP	CCATCATGAGTTCTTCACAAAAG TCGTCACGTCATCAGTGAGAC	350 bp	[29]

**Table 2:** Positive viral detections in plant and insect samples.

Plant		RT-PCR Positive Result	Insect		RT-PCR Positive Result
P1	*Brassica rapa*	TuYV	A1	*Brevicoryne brassicae*	TuYV
P2	*Chenopodium* sp.	-	A2	*Corytucha* sp.	-
P3	*Sonchus olearaceus*	-	A3	*Macrosiphum euphorbiae*	AMV, OPMV, ArLV
P4	*Urtica urens*	OPMV, TuYV	A4	*Microlophium carnosum*	AMV, TuYV
P5	*Epilobium* sp.	TuYV	A5	*Aulacorthum solani*	PLRV
P6	*Sonchus oleraceus*	-	A6	*Hyperomyzus lactucae*	PLRV
P7	*Sonchus oleraceus*	-	A7	*Hyperomyzus lactucae*	AMV
P8	*Sonchus oleraceus*	-	A8	*Uroleucon sonchi*	-
P9	*Amaranthus* sp.	TuYV	A9	*Macrosiphum euphorbiae*	-
P10	*Malva nicaeensis*	TuYV	A10	*Myzus persicae*	-
P11	*Sonchus oleraceus*	AMV, OPMV	A11	*Hyperomyzus lactucae*	OPMV, PLRV, cytorhabdovirus
P12	*Medicago sativa*	AMV	A12	*Chaitophorus leucomelas*	-
P13	*Medicago sativa*	AMV	A13	*Therioaphis trifolii*	-
P14	*Solanum tuberosum*	AMV	A14	*Aphis nerii*	PLRV
P15	*Solanum tuberosum*	AMV	A15	*Myzus persicae*	TuYV
P16	*Sonchus oleraceus*	AMV, TuYV	A16	*Brevicoryne brassicae*	TuYV
P17	*Chenopodium* sp.	AMV	A17	*Brevicoryne brassicae*	TuYV
P18	*Brassica rapa*	TuYV	A18	*Brevicoryne brassicae*	TuYV
P19	*Sonchus oleraceus*	Cytorhabdovirus, TuYV	A19	*Brevicoryne brassicae*	TuYV
P20	*Chenopodium* sp.	AMV	A20	*Brevicoryne brassicae*	TuYV
		-	A20.1	*Corythucha* sp.	ArLV
P21	*Brassica rapa*	AMV, TuYV	A21	*Brevicoryne brassicae*	-
P22	*Coriandrum sativum*	AMV, TuYV, OPMV	A22	*Cavariella* sp.	TuYV
P23	*Coriandrum sativum*	AMV, BtMV	A23	*Brevicoryne brassicae*	-
P24	*Coriandrum sativum*	AMV, BtMV	A24	*Myzus persicae*	TuYV
P25	*Coriandrum sativum*	AMV, BtMV	A25	*Cavariella* sp.	-
P26	*Coriandrum sativum*	AMV, TuYV, OPMV	A26	-	-
P27	*Coriandrum sativum*	AMV, BtMV	A27	-	-
P28	*Coriandrum sativum*	AMV, CeMV, TuYV, OPMV	A28	*Myzus persicae*	-
P29	*Coriandrum sativum*	AMV, CeMV	-	-	
P30	*Coriandrum sativum*	AMV	-	-	
P31	*Malva nicaeensis*	AMV	-	-	
P32	*Coriandrum sativum*	AMV	-	-	
P33	*Ocimum basilicum*	AMV	-	-	
P34	*Brassica rapa*	-	-	-	
P35	*Medicago sativa*	AMV	-	-	
P36	*Medicago sativa*	AMV	-	-	
P37	*Medicago sativa*	AMV, CeMV	-	-	
P38	*Amaranthus retroflexus*	AMV	-	-	

## Data Availability

Viral genomic sequences and SRA archives are available in GenBank under Bioproject number PRJNA1014719.

## Supplementary Materials

The following supporting information can be downloaded at: https://www.mdpi.com/article/10.3390/v16020226/s1, Figure S1: Neighbor-joining comparison among full genomes of *Solemoviridae* members identified in this study and reference isolates obtained from GenBank. Numbers in nodes represent bootstrap values obtained from 500 repetitions; Figure S2: Neighbor-joining comparison among full genomes of *Potyviridae* members identified in this study and reference isolates obtained from GenBank. Numbers in nodes represent bootstrap values obtained from 500 repetitions; Figure S3: Neighbor-joining comparison among full genomes of *Rhabdoviridae* members identified in this study and reference isolates obtained from GenBank. Numbers in nodes represent bootstrap values obtained from 500 repetitions; Figure S4: Conserved motifs of replicase proteins from the *Rhabdoviridae* family; Table S1: Primers used for complementary viral analyses; Table S2: Species identification for plants and insects collected during the survey.
