# Supplementary material for: Old and New Aphid-Borne Viruses in Coriander in Chile: An Epidemiological Approach
Source: Viruses. 2024 Jan 31;16(2):226. doi: 10.3390/v16020226 (PMC10893044; doi:10.3390/v16020226)

Figure S2: Neighbor joining comparison among full genomes of *Potyviridae* members identified in this study and reference isolates obtained from genbank. Numbers in nodes represent bootstrap values obtained from 500 repetitions.

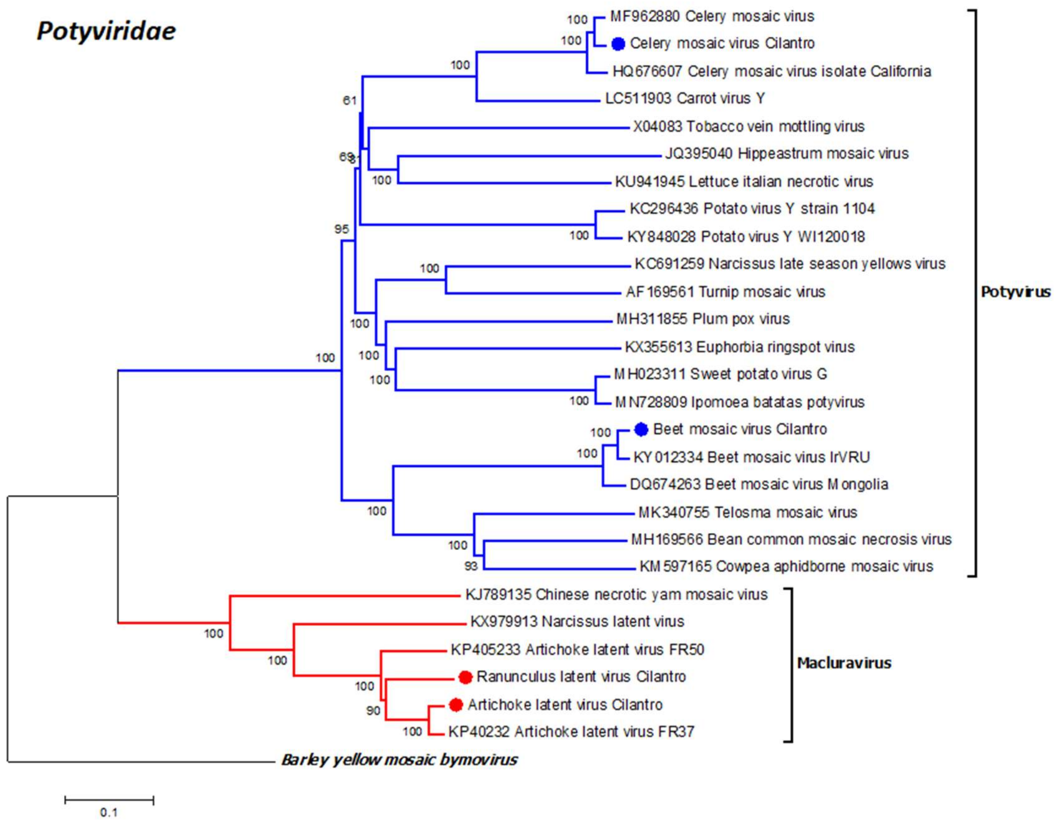

Supplement: Supplementary file 1 [file viruses-16-00226-s001.zip › Figure S2.pdf]
