# Supplementary material for: Old and New Aphid-Borne Viruses in Coriander in Chile: An Epidemiological Approach
Source: Viruses. 2024 Jan 31;16(2):226. doi: 10.3390/v16020226 (PMC10893044; doi:10.3390/v16020226)

Figure S4: Conserved motifs of replicase protein from *Rhabdoviridae* family

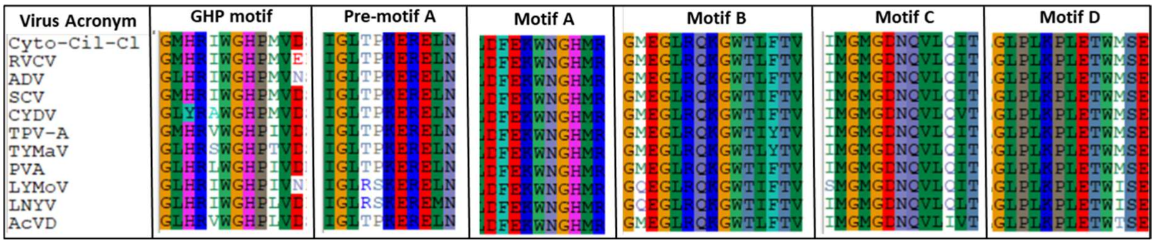

Supplement: Supplementary file 1 [file viruses-16-00226-s001.zip › Figure S4.pdf]
