# Supplementary material for: Old and New Aphid-Borne Viruses in Coriander in Chile: An Epidemiological Approach
Source: Viruses. 2024 Jan 31;16(2):226. doi: 10.3390/v16020226 (PMC10893044; doi:10.3390/v16020226)
Supplement: Supplementary file 1 [file viruses-16-00226-s001.zip › Table S1.pdf]

Table S1: Primers used for complementary viral analyses.

Virus	Genomic region	Sequence (5′-3′)	Amplicon size
PLRV	3′ terminal region	AGTGGTGCTTGATGAACATT<br>GCTACACAGTCGCGTCTTTC	982 bp
Cytorhabdovirus	L-Segment-Part1	CAGCGGCAAGAGATGAAGAC<br>AGGTCGAGGAGGGTATCTGT	1597 bp
	L-Segment-Part2	GCATTGATGTCCCACCTTTTG<br>TCAGTCGTCGATTGCATTGC	1607 bp
	L-Segment-Part3	ACTATCCAGAGACTTGCAATGC<br>GACACAGAGCGTCGACATTC	1589 bp
	L-Segment-Part4	TGTGCATGAAAGGATCGGAG<br>GTGGACCCTGACTTCCCTC	1684 bp
