# Supplementary material for: Old and New Aphid-Borne Viruses in Coriander in Chile: An Epidemiological Approach
Source: Viruses. 2024 Jan 31;16(2):226. doi: 10.3390/v16020226 (PMC10893044; doi:10.3390/v16020226)
Supplement: Supplementary file 1 [file viruses-16-00226-s001.zip › Table S2.pdf]

Table S2: Species identification for plants and insects collected during the survey.

						Insect Molecular identification		
Sampling period	Plot	Plant (sample code)	Plant identification	Plant associated insect (sample code)	Insect morphological identification	Molecular ID	Genbank reference number	Identity [a] (%)
M1	C1	P1	*Brassica rapa*	A1	*Brevicoryne brassicae*	*Brevicoryne brassicae*	MH407715.1	100
		P2	*Chenopodium* sp.	A2	*	*Corytucha padi*[b]	M63980941.1	86,8[c]
		P3	*Sonchus olearaceus*	A3	*Macrosiphum euphorbiae*	*Macrosiphum euphorbiae*	EU701726.1	100
		P4	*Urtica urens*	A4	*Microlophium carnosum*	*Microlophium carnosum*	JX507422.1	99,8
		P5	*Epilobium* sp.	A5	*Aulacorthum solani*	*Aulacorthum solani*	KF639123.1	99,8
		P6	*Sonchus oleraceus*	A6	*Hyperomyzus lactucae*	*Hyperomyzus lactucae*	MW596769.1	100
		P7	*Sonchus oleraceus*	A7	*Hyperomyzus lactucae*	*Hyperomyzus lactucae*	MW596769.1	100
		P8	*Sonchus oleraceus*	A8	*Uroleucon sonchi*	*Uroleucon sonchi*	MW596771.1	100
		P9	*Amaranthus* sp.	A9	*Macrosiphum euphorbiae*	*Macrosiphum euphorbiae*	EU701726.1	100
		P10	*Malva nicaeensis*	A10	*Myzus persicae*	*Myzus persicae*	KR889094.1	100
M2	C1	P11	*Sonchus oleraceus*	A11	*Hyperomyzus lactucae*	*Hyperomyzus lactucae*	MW596769.1	100
	C2	P12	*Medicago sativa*	A12	*nd*	*Chaitophorus leucomelas*	KF639289.1	99,84
		P13	*Medicago sativa*	A13	*Therioapihs* sp.	*Therioaphis trifolii*	MK766411.1	99,2
	C1	P14	*Solanum tuberosum*	A14	*Aphis nerii*	*Aphis nerii*	JF969254.1	99,84
		P15	*Solanum tuberosum*	A15	*Myzus persicae*	*Myzus persicae*	MT198936.1	99
	C3	P16	*Sonchus oleraceus*	A16	*Brevicoryne brassicae*	*Brevicoryne brassicae*	MH407715.1	100
		P17	*Chenopodium* sp.	A17	*Brevicoryne brassicae*	*Brevicoryne brassicae*	MH407715.1	100
		P18	*Brassica rapa*	A18	*Brevicoryne brassicae*	*Brevicoryne brassicae*	MH407715.1	100
		P19	*Sonchus oleraceus*	A19	*Brevicoryne brassicae*	*Brevicoryne brassicae*	MH407715.1	100
		P20	*Chenopodium* sp.	A20	*Brevicoryne brassicae*	*Brevicoryne brassicae*	MH407715.1	99
			Chenopodium sp.	A20.1	*	*Corythucha padi*[a]	M63980941.1	86,8[c]
		P21	*Brassica rapa*	A21	*Brevicoryne brassicae*	*Brevicoryne brassicae*	KT877996.1	100
	C4	P22	*Coriandrum sativum*	A22	*nd*	*Cavariella aegopodii*[a]	MG168203.1	97,1[c]
		P23	*Coriandrum sativum*	A23	*Brevicoryne brassicae*	*Brevicoryne brassicae*	KT877996.1	99,52
		P24	*Coriandrum sativum*	A24	*Myzus persicae*	*Myzus persicae*	MT198936.1	100
		P25	*Coriandrum sativum*	A25	*Cavariella aegopodii*	*Cavariella aegopodii*	GU667509.1	100
		P26	*Coriandrum sativum*	A26	nd	nd	-	-
		P27	*Coriandrum sativum*	A27	nd	*nd-*	-	-

		P28	*Coriandrum sativum*	A28	*Myzus persicae*	*Myzus persicae*	MT198936.1	99
		P29	*Coriandrum sativum*	-	-	-	-	-
M3	C5	P30	*Coriandrum sativum*	-	-	-	-	-
		P31	*Malva nicaeensis*	-	-	-	-	-
		P32	*Coriandrum sativum*	-	-	-	-	-
	C6	P33	*Ocimum basilicum*	-	-	-	-	-
	C3	P34	*Brassica rapa*	-	-	-	-	-
		P35	*Medicago sativa*	-	-	-	-	-
		P36	*Medicago sativa*	-	-	-	-	-
		P37	*Medicago sativa*	-	-	-	-	-
	C1	P38	*Amaranthus retroflexus*	-	-	-	-	-
